# Novel sporadic and recurrent mutations in *KRT5* and *KRT14* genes in Polish epidermolysis bullosa simplex patients: further insights into epidemiology and genotype–phenotype correlation

**DOI:** 10.1007/s13353-015-0310-9

**Published:** 2015-10-02

**Authors:** K. Wertheim-Tysarowska, M. Ołdak, A. Giza, A. Kutkowska-Kaźmierczak, J. Sota, D. Przybylska, K. Woźniak, D. Śniegórska, K. Niepokój, A. Sobczyńska-Tomaszewska, A. M. Rygiel, R. Płoski, J. Bal, C. Kowalewski

**Affiliations:** Department of Medical Genetics, Institute of Mother and Child, Kasprzaka 17a, 01211 Warsaw, Poland; Department of Histology and Embryology, Center of Biostructure Research, Medical University of Warsaw, Chalubinskiego 5, 02004 Warsaw, Poland; Department of Dermatology and Immunodermatology, Medical University of Warsaw, Chalubinskiego 5, 02004 Warsaw, Poland; Department of Genetics, Medical University of Warsaw, Pawińskiego 5a, 02004 Warsaw, Poland; Department of Genetics, World Hearing Center, Institute of Physiology and Pathology of Hearing, Warsaw, Poland

**Keywords:** *KRT5*, *KRT14*, Epidermolysis bullosa simplex (EBS)

## Abstract

Epidermolysis bullosa simplex (EBS) is a hereditary genodermatosis characterised by trauma-induced intraepidermal blistering of the skin. EBS is mostly caused by mutations in the *KRT5* and *KRT14* genes. Disease severity partially depends on the affected keratin type and may be modulated by mutation type and location. The aim of our study was to identify the molecular defects in *KRT5* and *KRT14* in a cohort of 46 Polish and one Belarusian probands with clinical suspicion of EBS and to determine the genotype–phenotype correlation. The group of 47 patients with clinical recognition of EBS was enrolled in the study. We analysed all coding exons of *KRT5* and *KRT14* using Sanger sequencing. The pathogenic status of novel variants was evaluated using bioinformatical tools, control group analysis (DNA from 100 healthy population-matched subjects) and probands’ parents testing. We identified mutations in 80 % of patients and found 29 different mutations, 11 of which were novel and six were found in more than one family. All novel mutations were ascertained as pathogenic. In the majority of cases, the most severe genotype was associated with mutations in highly conserved regions. In some cases, different inheritance mode and clinical significance, than previously reported by others, was observed. We report 11 novel variants and show novel genotype–phenotype correlations. Our data give further insight into the natural history of EBS molecular pathology, epidemiology and mutation origin.

## Introduction

Epidermolysis bullosa simplex (EBS) is a rare hereditary genodermatosis characterised by intraepidermal blistering of the skin upon mild trauma (Fine 2010). Several subtypes of EBS have been described according to clinical, ultrastructural and molecular findings. The most frequent variants are: EBS, localised (EBS-loc) with blistering confined to the hands and feet, EBS-generalised intermediate (EBS-gen intermed, formerly non-Dowling-Meara or EBS-gen nDM), in which blistering occurs on the whole body area, and more severe: EBS-generalised severe (EBS-gen sev, formerly Dowling-Meara or EBS-gen DM), where mucosa is also involved (Coulombe and Lee 2012; Fine et al. 2008). In more than 75 % of cases, EBS is caused by mutations in the *KRT5* and *KRT14* genes, affecting keratin 5 (K5) or keratin 14 (K14), respectively (Bolling et al. 2011); however, mutations in nine other genes were reported to cause the EBS phenotype (Fine et al. 2014).

EBS can be inherited in autosomal dominant or recessive mode. More than 86 % of mutations occurring in *KRT5* or *KRT14* are dominantly acting missenses (Coulombe and Lee 2012; Fine et al. 2008). Regardless of the keratin type affected, most of these mutations change amino acid residues in the central L-helical rod domain, leading to more severe EBS phenotypes. In contrast, milder EBS is often caused by mutations in the *K5* located in non-helical linker regions and in the head domain (Coulombe and Lee 2012). In addition, the severity of the disease is further modulated by the location of substituted amino acid within repetitive motif [i.e. heptad structure (abcdefg)_n_] of the helical domain. According to the superhelix model, the phenotype caused by substitutions of amino acids directly involved in the interaction between heterodimeric keratins is more severe compared to those affecting other residues (Pauling and Corey 1953; Müller et al. 2006). Furthermore, other data indicate that mutation dosage and biophysical properties of introduced amino acids are also of importance with regards to exacerbation of the EBS severity (Shinkuma et al. 2013; Ołdak et al. 2011).

### Objective

The aim of our study was to identify the molecular defects in the *KRT5* and *KRT14* genes in a cohort of 46 Polish and one Belarusian probands with clinical suspicion of EBS and to determine the genotype–phenotype correlations.

## Materials and methods

### Study subjects

The cohort of 47 EBS probands from 47 families and their family members were enrolled in the study. EBS was diagnosed based on clinical symptoms (according to the consensus from 2014; Fine et al. 2014) and/or of skin biopsies results. DNA isolated from 100 healthy population-matched subjects was used as control samples.

All participants gave formal consent for their participation in the study. The study was approved by the local ethics committee.

### Mutation analysis

The mutation analysis of *KRT5* and/or *KRT14* was performed using Sanger sequencing. Primers for *KRT14* were as described previously (Ołdak et al. 2010) and for *KRT5* self-designed in PRIMER3 (available on request). Fluorochromatograms were analysed in Mutation Surveyor software using NM_000526 and NM_000424 as references for *KTR14* and *KRT5*, respectively. The control group was screened for each of the novel mutations identified by us. The mutation segregation in the family was performed for 27 out of 38 families. For the remaining patients, the DNA from their parents and/or other relatives was not available.

### In silico analysis

The Clustal X analysis was performed to check evolutionary conservation of novel mutations, the SSF (Splicing Sequences Finder), NNSPLICE (Splice Site Prediction by Neural Network) and HSF (Human Splicing Finder) softwares were used to evaluate in silico the potential effect of novel variants on splicing aberration and PolyPhen-2 to evaluate protein structure distortion.

## Results

### Genotyping results

In 38/47 probands, we detected mutations in either *KRT5* or *KRT14* and, in one case, we found mutations in both genes. In total, we identified 29 different mutations, i.e. 16 in *KRT5* and 13 in *KRT14*, 11 of them were novel and six were recurrent (*KRT5*: p.Glu170Lys, p.Leu325Phe, p.Val186Met; *KRT14*: p.Arg125His, p.Met272Thr, p.Val133Met). Genotyping results, mutations details and the family segregation results are given in Table 1.

**Table 1:** Results of the molecular analysis of Polish patients with epidermolysis bullosa simplex (EBS)

Number	Gene	EBS subtype	Genotype traditional	Genotype HGVS	Exon	Domain	Heptad	Inheritance
1	*KRT14*	EBS-gen sev	p.Tyr129Asp/-	c.[385T>G];[=]	1	1a	d	de novo
2	*KRT14*	EBS-gen intermed	p.Val133Met/-	c.[397G>A];[=]	1	1a	a	AD
3	*KRT14*	EBS-gen sev	p.Met119Thr/-	c.[356T>C];[=]	1	1a	a	n.d.
4	*KRT14*	n.d.	p.Glu411del/-	c.[1231_1233delGAG];[=]	6	2b	g	n.d.
5	*KRT14*	n.d.	p.Arg388Cys/-	c.[1162C>T];[=].	7	2b	e	AD
6	*KRT14*	n.d.	p.Arg125His/-	c.[374G>A];[=]	1	1a	g	AD
7	*KRT14*	EBS-gen sev	p.Arg125His/-	c.[374G>A];[=]	1	1a	g	AD
8	*KRT14*	EBS-loc	p.Val133Met/-	c.[397G>A];[=]	1	1a	a	AD
9	*KRT14*	EBS-gen sev	p.Arg125Cys/-	c.[373C>T];[=]	1	1a	g	de novo
10	*KRT14*	n.d.	**p.Arg125Leu/-** ^a^	**c.[374G>T]**;[=]	1	1a	g	de novo (?)
11	*KRT14*	EBS-loc	**p.Val270Ala/-** ^b^	**c.[809T>C]**;[=]	4	l12	n.a.	de novo
12	*KRT14*	EBS-loc	p.Ala413Thr/-	c.[1237G>A];[=]	6	2b	b	n.d.
13	*KRT14*	EBS-loc	p.Val133Met/-	c.[397G>A];[=]	1	1a	a	n.d.
14*	*KRT14*	EBS-gen sev	p.Asn123Ser/-	c.[368A>G];[=]	1	1a	e	de novo
15	*KRT14*	EBS-loc	p.Met272Thr/-	c.[815T>C];[=]	4	l12	n.a.	AD
16	*KRT5, KRT14*	EBS-loc (!)	*KRT5*: **p.Arg471His/-**; *KRT14*: p.Met272Thr/-^c^	NM_000424:**c.[1412G>A]**;[=]; NM_000526:c.[815T>C];[=]	7 4	2b l12	e n.a.	AD
17	*KRT14*	EBS-loc/gen	**p.Leu418Gln/-** ^d^	**c.[1253T>A];[=]**	6	2b	g	AD
18	*KRT14*	n.d.	p.Met272Thr/-	c.[815T>C];[=]	4	l12	n.a.	AD
19	*KRT5*	n.d.	**p.Leu203Met/-**	**c.[607C>A]**;[=]	2	1a	d	AD
20	*KRT5*	EBS-gen intermed	p.Val186Met/-	c.[556G>A];[=]	2	1a	a	AD
21	*KRT5*	EBS-gen intermed	p.Val186Met/-	c.[556G>A];[=]	2	1a	a	AD
22	*KRT5*	EBS-loc	p.Glu170Lys/-	c.[508G>A];[=]	1	1a	f	AD (partial penetration)
23	*KRT5*	EBS-loc	**p.Leu325Phe/-**	**c.[973C>T]**;[=]	2	l12	n.a.	AD
24	*KRT5*	EBS-gen sev	**p.Thr144_Val145del/-**	**c.[431_436delCTGTCA]**;[=]	1	head	n.a.	de novo
25	*KRT5*	EBS-loc	**p.Asn146Tyr/-**	**c.[436A>T]**;[=]	1	head	n.a.	AD
26	*KRT5*	n.d.	**c.556-2A>G/-**	**c.[556-2G>A]**;[=]	2	1a	n.a.	AD
27	*KRT5*	EBS-loc	p.Glu170Lys/-	c.[508G>A];[=]	1	1a	f	(?)
28	*KRT5*	EBS-gen intermed	p.Val143Ala/Glu170Lys	c.[428T>C];[508G>A]	1	head/1a	f	AR
29	*KRT5*	n.d.	p.Leu325Phe/-	c.[973C>T];[=]	5	l12	n.a.	AD
30	*KRT5*	EBS-gen intermed	p.Glu170Lys/Glu170Lys^e^	c.[508G>A];[508G>A]	1	1a	f	AD (partial penetration)
31	*KRT5*	n.d.	p.Glu190Lys/-	c.[568G>A];[=]	2	1a	e	AD
32	*KRT5*	n.d.	p.Val143Phe/-	c.[427G>T];[=]	1	head	n.a.	de novo (?)
33	*KRT5*	n.d.	**p.Glu477Gly/-** ^f^	**c.[1430A>G]**;[=]	7	2b	d	n.d.
34	*KRT5*	EBS-loc (!!)	p.Gly476Asp/-^g^	c.[1427G>A];[=]	7	2b	c	AD
35	*KRT5*	EBS-MP	p.Pro25Leu/-^f^	c.[74C>T];[=]	1	head	n.a.	n.d.
36	*KRT5*	EBS-loc	p.Leu325Pro/-^f^	c.[974T>C];[=]	2	l12	n.a.	n.d.
37	*KRT5*	EBS-loc	p.Glu170Lys/-	c.[508G>A];[=]	1	1a	f	n.d.
38	*KRT5*	n.d.	**p.Tyr470Ter/-**	**c.[1410C>G]**;[=]	7	2b	d	de novo
39–47	*KRT5, KRT14*	n.d.	-/-	c.[=];[=]	n.a.	n.a.	n.a.	n.d.

(!) EBS-loc in heterozygous members of the family (genotyped as p.Met272Thr/-); (!!) EBS-gen intermed seen in one family member; *n.a.* not applicable; *n.d.* no data; *AD* autosomal dominant; *AR* autosomal recessive; (?) de novo event is suggested based on family history, but no molecular confirmation has been performed. Novel mutations are in **bold**

*Patient of Belarusian origin

The letters in superscript refer to the following references with profound description of our case: ^a^ Ołdak et al. (2010); ^b^ Ołdak et al. (2013); ^c^ Wertheim-Tysarowska et al. (2014); ^d^ Jankowski et al. (2014); ^e^ Ołdak et al. (2011); ^f^ Hamada et al. (2005); ^g^ Kowalewski et al. (2009)

### In silico characteristics of novel mutations

Alignment of epidermal keratins type I and II showed that novel missenses and in frame deletion localise in highly conserved regions of K5 or K14 proteins. All missenses were assigned as ‘probably pathogenic’ by the PolyPhen-2 algorithm. Total abolition of the intron 1 acceptor site in *KRT5* by c.556-2A>G was indicated unanimously, which, according to in silico predictions, leads to aberrant pre-mRNA splicing and production of a truncated protein. No mutations in *KRT5* and *KRT14* were found in the control group.

## Discussion

Although EBS has heterogenic genetic background, *KRT5* and *KRT14* are the most important genes in the development of this disorder and EBS can still be regarded as a model keratinopathy.

We identified mutations in *KRT5* or/and *KRT14* genes in 38 unrelated patients; that is, on one of the largest published groups of patients (Bolling et al. 2011; Jerábková et al. 2010; Chen et al. 1995; Pfendner et al. 2005; Arin et al. 2010). Although around 120 mutations in *KRT5* and 95 in *KRT14* genes were published so far and patients of Central European origin were also included in other reports, we not only identified recurrent mutations but were also able to find unique and novel ones.

All recurrent mutations from our group, except for p.Leu325Phe in *KRT5*, were previously described (Table 2). In contrast to the widely known hot spot of *KRT14* codon 125, in which six different amino acid substitutions were described in over 67 patients worldwide, the origin of the other mutations is less clear (Human Intermediate Filament Database; Szeverenyi et al. 2008; Rugg et al. 2007). According to published data, the p.Glu170Lys in *KRT5* and p.Met272Thr in *KRT14* were mostly found in patients originating from Central Europe, which may strongly suggest common ancestry. The p.Val133Met was reported previously in two Scottish families (Rugg et al. 2007). The data about these families are highly limited, but due to centuries of close relations between Poland and Scotland, we cannot rule out that, in this case, common origin is a clue once again. However, the fact that two other amino acid substitutions of Val 133 are known, of which the p.Val133Leu is seemingly more common in Europe, we cannot rule out that codon 133 is prone to mutational events. Finally, the p.Val186Met, which was found in distinct populations (Turkey, Japan and Poland) and also represents only one out of three different valine 186 amino acid substitutions, seems to be the probable mutational hot spot (Arin et al. 2010; Hattori et al. 2006; Yasukawa et al. 2006).

**Table 2:** Summary of patients with selected recurrent mutations in *KRT5* and *KRT14* genes identified by us and others

	Genotype - cDNA name	Genotype - Protein name	Country of origin	Number of unrelated patients	Mutation origin	Ref.
*KRT5*	c.[508G>A];[=]	**p.[Glu170Lys];[=]**	Germany	1	F	Müller et al. (2006)
			Hungary	1	F	Glász-Bóna et al. (2009)
			Czech Republic	1	F	Jerábková et al. (2010)
			China	1	F	Tang et al. (2009)
			Japan	1	F	Yasukawa et al. (2006)
			Poland	1	F	This work
			Poland	2	unknown	
	c.[428T>C];[508G>A]	p.[Val143Ala];**[Glu170Lys]**	Poland	1	F	
	c.[508G>A];[508G>A]	p.[Glu170Lys];**[Glu170Lys]**	Poland	1	F	
			Total	10		
	c.[556G>A];[=]	**p.[Val186Met];[=]**	Turkey	1	F	Arin et al. (2010)
			Japan	1	de novo	Hattori et al. (2006)
			Japan	1	F	Yasukawa et al. (2006)
			Poland	2	F	This work
			Total	5		
*KRT14*	c.[815T>C];[=]	**p.[Met272Thr];[=]**	Germany	4	F	Arin et al. (2010)
			Germany	1	de novo	Müller et al. (2006)
			Poland	3	F	This work
			Total	8		
	c.[397G>A];[=]	**p.[Val133Met];[=]**	Scotland	2	F	Rugg et al. (2007)
			Poland	2	F	This work
			Poland	1	unknown	
			Total	5		

Recurrent mutations are bolded

*F* familial

We also identified two families with p.Leu325Phe in *KRT5*. We cannot speculate about the origin of this mutation, since we were unable to test whether both families share ancestry. Noteworthy, another mutation of codon 325 (p.Leu325Pro) has also been found and, therefore, we cannot excluded that this codon is another one which is prone to mutational events and these mutations arose independently (Sørensen et al. 1999; Hamada et al. 2005).

The correlation between phenotype and genotype can only be performed when the EBS subtype is recognised on the basis of clinical symptoms, which tend to change over time. Therefore, in some patients, it is not possible to make a proper diagnosis during early childhood. This explains mostly the reason why, in some probands, we could not determine the EBS subtype. However, the other important issue was that some of them had dermatological consultation once or twice only, making proper subtype distinction highly tentative.

It is generally accepted that mutations in *KRT5* are, overall, associated with milder clinical outcome and that the location of mutation within the most conserved regions of *K5* and *K14* is linked to a more severe clinical picture. We also observe such tendencies in our heterozygous patients. Most of our mutations (16 vs. 13) were located in the *KRT5* gene, which is in accordance with the fact that patients were predominantly EBS-loc. Furthermore, 90 % (8/9) of mutations identified in patients with generalised EBS subtypes (severe and intermediate) are localised in helical segment 1a or 2b of either *K5* or *K14*, while far fewer, 50 % (6/12), of mutations found in localised EBS patients share this location. Furthermore, our results are also in agreement with the model of Liovic et al. (2001): the precise location of a given amino acid within heptad repetitive sequence (abcdefg)n modulates further the disease severity. Amino acids at positions a/d and e/g are directly involved in maintenance and stabilisation of the K5/K14 heterodimer; thus, their substitutions are more deleterious (Smith et al. 2004; Müller et al. 2006). Indeed, 8/8 (100 %) of mutations localised in the 1a segment in patients with generalised EBS subtypes are situated in these positions. On the contrary, only 33 % (2/6) of mutations found in 1a or 2b segments in EBS-loc patients were identified in position ‘a.’ These observations are highly encouraging with respect to phenotype prediction purposes; however, a closer look reveals a possible engagement of other modulatory factors (e.g. sequence variations in other alleles or genes encoding other keratins).

**Fig. 1:**
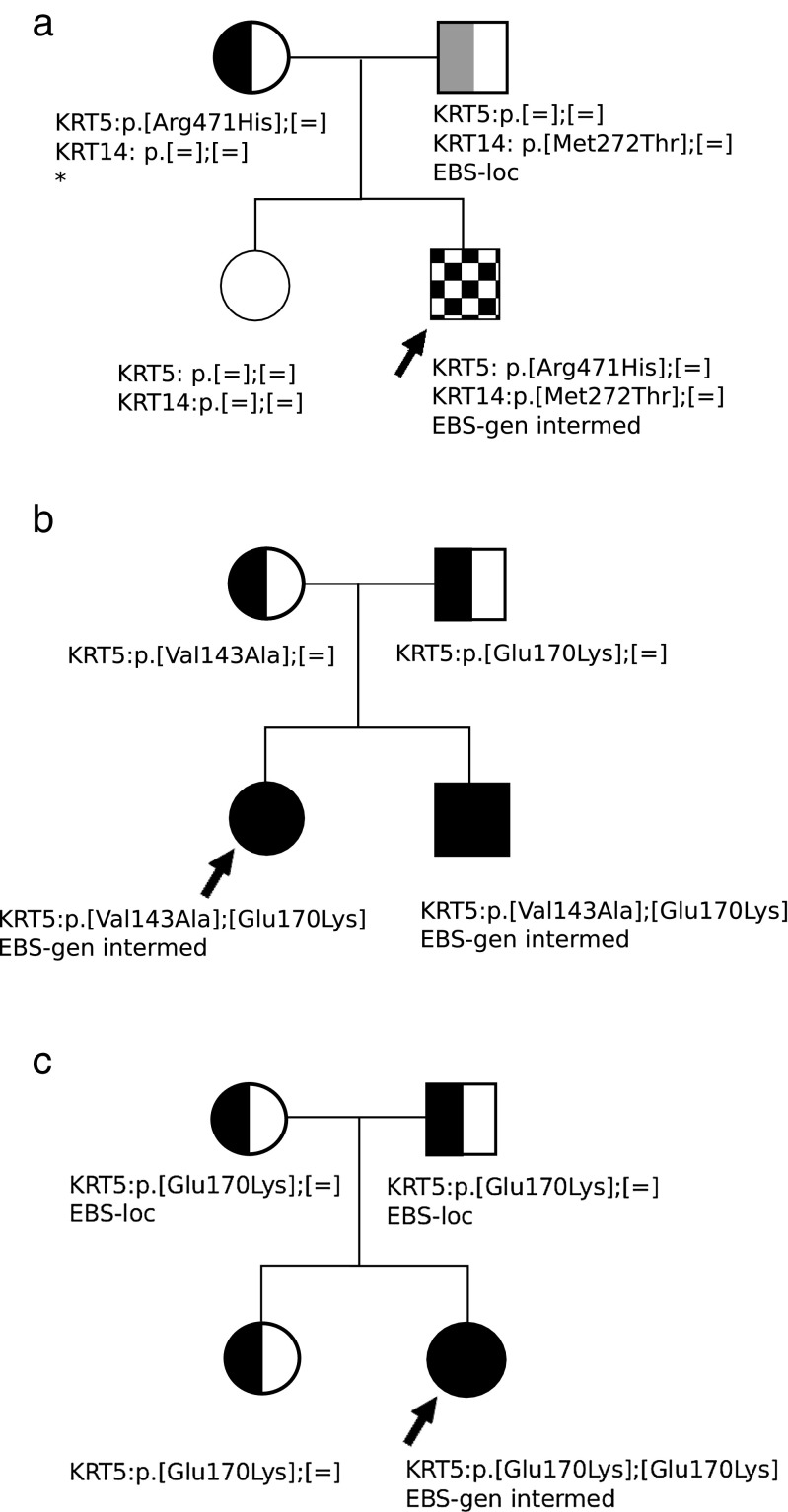
Pedigrees of families 16 (**a**), 28 (**b**) and 30 (**c**) showing probands and their first-degree relatives. Symbols: *half-black* heterozygous mutation in *KRT5*; *solid black* mutation in both alleles of *KRT5*; *half-grey* heterozygous mutation in *KRT14*; *checkered pattern* mutation in one allele of *KRT5* and in one allele of *KRT14*; *=* no mutation detected in single allele; * feet skin fragility, but the final diagnosis of epidermolysis bullosa (EB) and EB type/subtype has not been confirmed clinically (**a**: modified from Wertheim-Tysarowska et al. 2014; **c**: modified from Ołdak et al. 2011)

The most frequent mutation, p.Glu170Lys in *KRT5*, according to our findings and data published by others can have various clinical consequences (Ołdak et al. 2010; Müller et al. 2006; Jerábková et al. 2010; Arin et al. 2010; Yasukawa et al. 2002). This substitution occurred in five probands of our group. In three EBS-loc patients, it was present in one allele and no other mutations in *KRT5* or *KRT14* were detected. However, in two remaining patients with EBS-gen intermed, the p.Glu170Lys was found in both alleles or in a heterozygous state with p.Val143Ala. The first case has already been reported by Ołdak et al. (2011), who found symptoms of localised EBS in both heterozygous (p.Glu170Lys/-) parents of the patient. Interestingly, in the proband’s older sister, despite the same genotype (p.Glu170Lys/-), no clinical symptoms were observed (Fig. 1). In the second family, the proband and her EBS-gen intermed brother were both genotyped as compound heterozygotes p.Glu170Lys/p.Val143Ala (Fig. 1). Both parents were proved to be carriers of p.Glu170Lys (father) or p.Val143Ala (mother), and none of them reported any clinical signs of EBS. Of note, the p.Val143Ala mutation was previously described in the literature, in two patients with autosomal dominant EBS-loc (Jerábková et al. 2010). Why some p.Glu170Lys and p.Val143Ala heterozygotes do have clinical symptoms of EBS-loc and others do not remains an open question. According to our knowledge, there is only one another patient, reported by Yasukawa et al. (2002), with the p.Glu170Lys mutation in compound heterozygosity with the other mutation (p.Glu418Lys) in *KRT5*. In this case, clinical symptoms and also keratin clumping during in vitro assay were aggravated when both mutations were present. However, in this case, the p.Glu170Lys without p.Glu418Lys was identified in family members with milder disease. Furthermore, we found EBS-loc and EBS-gen intermed ratio 1:1 in one large family with p.Leu418Gln in *KRT14* and identified a family with digenic *KRT5* and *KRT14* EBS (Fig. 1; Wertheim-Tysarowska et al. 2014; Jankowski et al. 2014).

Another example is p.Leu325Phe in *KRT5*, where we have also encountered some difficulties in establishing its pathogenic status and phenotypic effect. It was identified in the EBS-loc patient, her affected father and grandfather. The grandfather claimed to be healthy, but admitted his mother (the proband’s great grandmother, deceased, not analysed) had clinical symptoms resembling EBS. Since the grandfather did not agree for clinical evaluation, we only had limited data available, but managed to establish that slight skin changes (e.g. keratoderma) were present. Furthermore, we also detected the p.Leu325Phe in the other EBS-loc family, where, in contrast, we were able to show its cosegregation with the EBS phenotype (six affected patients) and prove its pathogenic status.

Overall, our observations regarding genotype–phenotype correlations have important impact on genetic counselling, indicating that offspring phenotype can vary from parental clinical outcome and that full sequencing of both keratins should be considered in the case of the EBS patient’s partner.

In about 17 % of our probands, particularly in generalised EBS cases, mutations were due to de novo events (or resulted from a germinal mosaicism). This number is lower than that reported by others (Bolling et al. 2011; Jerábková et al. 2010; Pfendner et al. 2005), but still indicates the high rate of spontaneous events. Nevertheless, we have also shown that parent testing is indispensable in order to exclude the possibility of being an asymptomatic carrier or having discrete EBS phenotype. Therefore, we also suggest to perform analysis of the whole coding region of *KRT5* and *KRT14* in patients whose phenotype is more severe than that observed in other family members.

Although EBS is the most frequent type of EB, recent findings prove that there are still a lot of unanswered questions regarding natural history of the disease and its genetic background (Hamada et al. 2013). Molecular analyses performed worldwide indicate that *KRT5* and *KRT14* mutations are responsible for the majority of EBS and can be found in 70–75 % of patients (Bolling et al. 2011; Rugg et al. 2007). Thus, the detection rate observed by us in this study (80 %) is in agreement with the results obtained by others.

It should be noted however, that another skin disorder, acral peeling skin syndrome (APSS, caused by mutations in *TGM5*), has only recently been classified as another subtype of EBS (Fine et al. 2014). Therefore, when all Polish probands with APSS (*n* = 20; for further details, see Szczecinska et al. 2014) and other types of EBS (*n* = 47; this study) are considered (*n* = 20 + 47 = 67), the numbers change as follows: *KRT5* and *KRT14* mutations are present in 38/67 (57 %) of total EBS probands and mutations in *TGM5* in 20/67 (30 %) probands. Hence, analysis of these three genes gives a total detection rate of 87 % in the Polish population of EBS patients classified according to current recommendations.

The lack of mutations in *KRT5* and *KRT14* in nine patients (who were also negative for *TGM5* mutations, data not shown) may indicate an existence of large rearrangements or mutations either in intronic/regulatory elements of *KRT14*, *KRT5* and *TGM5* or, more likely, in the other genes. Indeed, EBS is the most heterogenic type of EB and has recently been proved to be caused by mutations in at least eight other genes besides *KRT5*, *KRT14* and *TGM5*, which are: *PLEC*, *PKP1*, *DSP*, *JUP*, *DST*, *EXPH5*, *ITGA6* and *ITGB4* (Fine et al. 2014). Extended molecular analyses are planned in the future.

In summary, our data provide further insight into EBS molecular pathology, natural history and epidemiology: we provide novel evidence that more mutations than we thought earlier can have variable clinical significance and give more data concerning the origin of several mutations. Our results are of particular importance for genetic counselling and prognostic purposes, and have practical implication in diagnostics.
